# Invasive Breast Carcinoma With a Multi-cystic Papillary Growth Pattern: A Unique Morphology of Invasion Not Currently Well Classified by the World Health Organization

**DOI:** 10.7759/cureus.94223

**Published:** 2025-10-09

**Authors:** Evan K Tweed, Andrew J Berman, Michelle Josey

**Affiliations:** 1 Department of Pathology, Brooke Army Medical Center, San Antonio, USA

**Keywords:** breast papillary lesions, encapsulated papillary carcinoma, invasive breast carcinoma, invasive ductal carcinoma of the breast, invasive papillary carcinoma

## Abstract

Papillary neoplasia of the breast is notoriously difficult to classify, even for seasoned pathologists and those subspecialized in breast pathology. Over the past few decades, the classification of these lesions has undergone multiple updates, and several newly recognized entities have been added. Multiple benign and malignant breast entities are characterized by the World Health Organization (WHO). Despite these categorizations, pathologists are sometimes presented with papillary morphologies and features that do not fit perfectly into any one of these classifications. We present a case of a 62-year-old female with an invasive breast carcinoma of a type that we believe is not currently well characterized by the WHO and that exhibits a papillary growth pattern, specifically invading as multi-cystic papillary structures.

## Introduction

Papillary neoplasia is characterized by a proliferation of fibrovascular cores lined by epithelium. Papillary lesions of the breast are either intraductal or invasive. Benign intraductal papillary neoplasms have a continuous layer of myoepithelial cells at the periphery and within their fibrovascular cores, whereas malignant papillary lesions show variable distributions of myoepithelial cells at the periphery and along their papillae. Unlike conventional mammary carcinomas, the epithelial component determines whether a papillary lesion is benign, atypical, or malignant [1,2]. According to the World Health Organization (WHO) classification of breast tumours, fifth edition, the papillary neoplasms of the breast include intraductal papilloma, papilloma with atypical ductal hyperplasia (ADH) or ductal carcinoma in situ (DCIS), papillary DCIS, encapsulated papillary carcinoma (EPC), EPC with invasion, solid papillary carcinoma (SPC), invasive SPC, SPC with invasion, and invasive papillary carcinoma (IPC) [1]. Tall cell carcinoma with reverse polarity is an extremely rare entity showing a solid papillary pattern and is classified by the WHO under rare and salivary gland-type tumors [1,3]. Metastases from papillary thyroid carcinoma, pulmonary papillary adenocarcinoma, and ovarian serous carcinoma are also diagnostic considerations when evaluating a papillary breast lesion [4]. In this case report, we present a breast carcinoma with a distinctive multicystic papillary growth pattern that may represent either a distinct entity or a unique, unrecognized subtype of IPC or EPC.

## Case presentation

**Figure 1:**
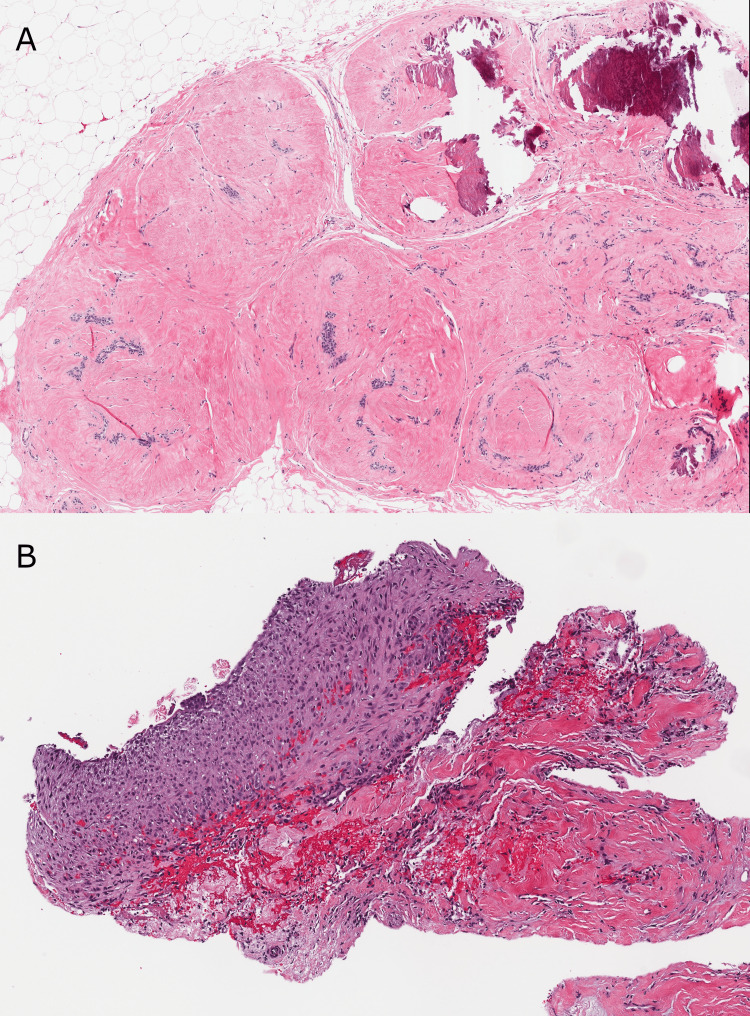
Prior core biopsies demonstrating benign findings only. (A) H&E - Biphasic (epithelial and stromal) proliferation of breast tissue with stromal proliferation outgrowing the epithelial proliferation, causing an intracanalicular pattern of growth, consistent with fibroadenoma (20x). (B) H&E - Multilayered epithelial cyst lining with associated lymphohistiocytic inflammation and underlying fibrosis, consistent with cyst or duct rupture (40x). Slides from the patient's bilateral breast biopsies that were performed in 2003 and demonstrated benign breast tissue are not available for review and are not pictured.

**Figure 2:**
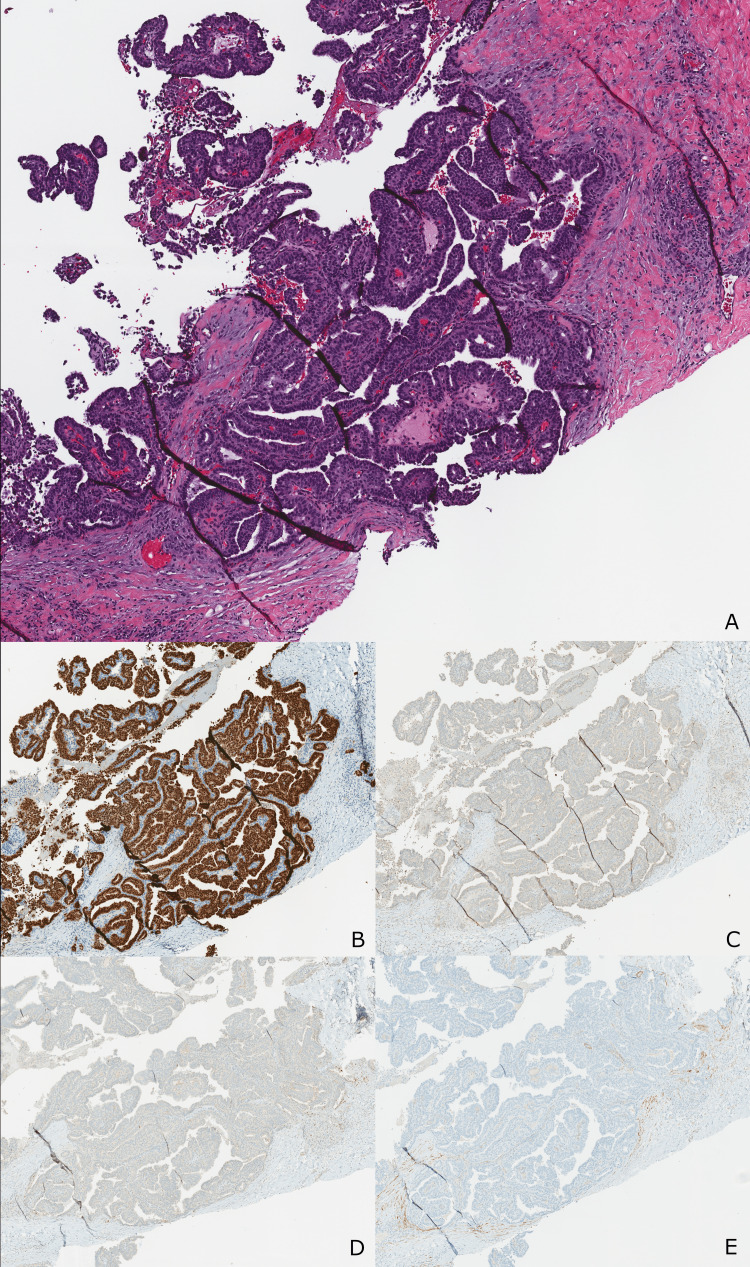
Core biopsy demonstrating atypical cells with papillary architecture. (A) H&E - Atypical epithelial cells with low to intermediate grade nuclei forming papillary projections with well-defined fibrovascular cores (40x). (B) The nuclei of the atypical cells stain strongly and diffusely positive for ER immunostain (40x). (C) The atypical cells demonstrate loss of the normal (cytoplasmic) staining pattern of CK5/6 immunostain (40x). (D) p63, a nuclear immunostain (40x). (E) Calponin, a cytoplasmic immunostain, demonstrates the absence of intralesional and peripheral myoepithelial cells (40x).

The patient was a 62-year-old, multiparous, postmenopausal, African American female who reported a lengthy and continuous history of painful and tender breasts and had undergone multiple biopsies. Bilateral breast biopsies in 2003 demonstrated benign breast tissue; a left breast biopsy in 2016 was found to be consistent with a fibroadenoma (Figure 1A), and a right breast biopsy in 2021 demonstrated inflammatory changes suggesting cyst or duct rupture (Figure 1B). In 2023, targeted sonography of a locus of pain in the upper outer right breast demonstrated a 3.5 × 2.0 × 1.7 cm irregularly shaped, Breast Imaging-Reporting and Data System (BIRADS) category 4C, complex cystic and solid mass at the 10 o’clock position, 6 cm from the nipple and near the clip from a prior biopsy. Ultrasound-guided targeted core biopsy of the lesion was performed, and subsequent histologic examination revealed cystic spaces with papillary projections composed of fibrovascular cores covered by atypical epithelial cells of low to intermediate nuclear grade (Figure 2A). The atypical cells were diffusely estrogen receptor positive and negative for cytokeratin 5/6 (Figure 2B,C). Lack of p63 and calponin staining demonstrated the absence of a myoepithelial component within the papillae and at the periphery of each cystic papillary structure (Figure 2D,E). This biopsy specimen was signed out as “papillary carcinoma, at least in situ, intermediate grade,” in which EPC was favored, but definitive classification was left to a resection specimen. The follow-up hormonal biomarkers were estrogen receptor (ER) positive (91-100%) and progesterone receptor (PR) positive (51-60%). Human epidermal growth factor receptor 2 (HER2) and Ki-67 testing were not performed on the biopsy specimen.

**Figure 3:**
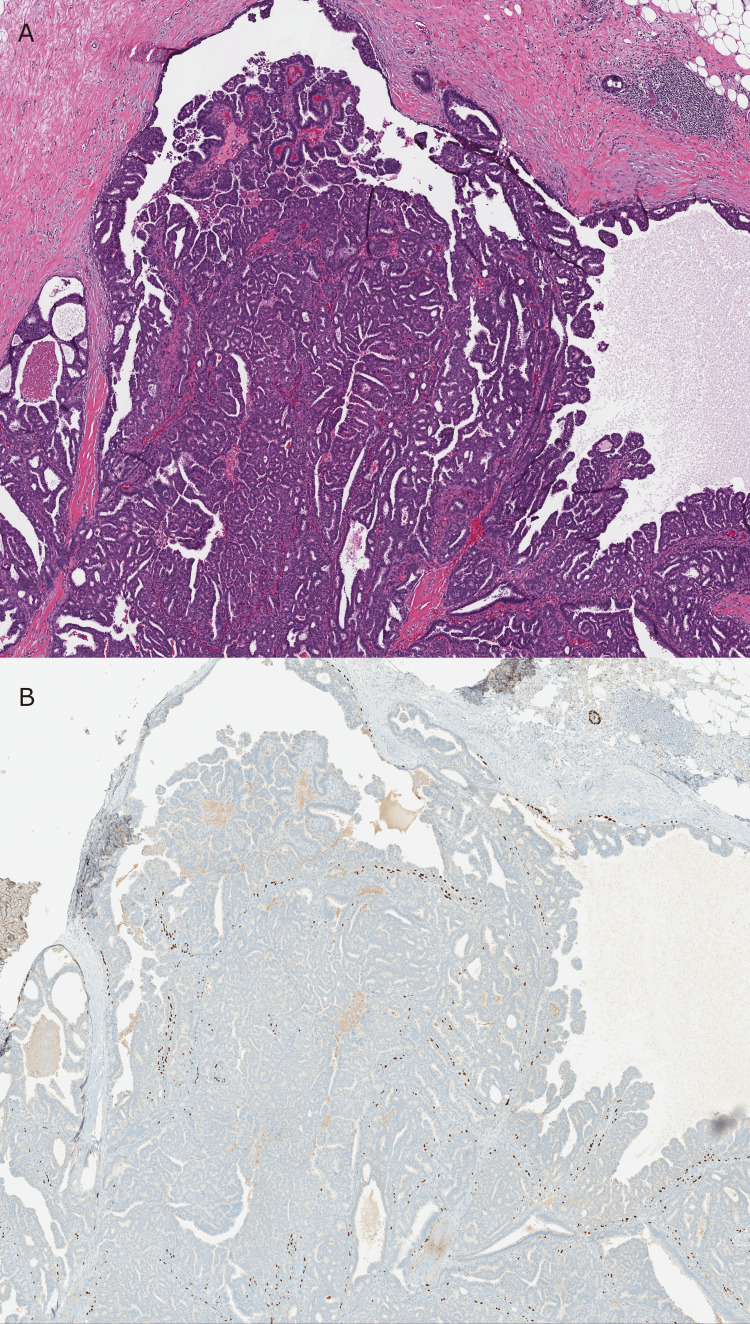
Area from the mastectomy specimen demonstrating atypia/DCIS involving a papilloma. (A) H&E - Low-power view of an extensive papillary proliferation of cells with low to intermediate grade atypia. The fibrovascular cores are well-defined (20x). (B) p63 immunostain demonstrates nuclear staining in peripheral and intralesional myoepithelial cells, supporting that this represents a papilloma involved by atypia/DCIS (20x).

**Figure 4:**
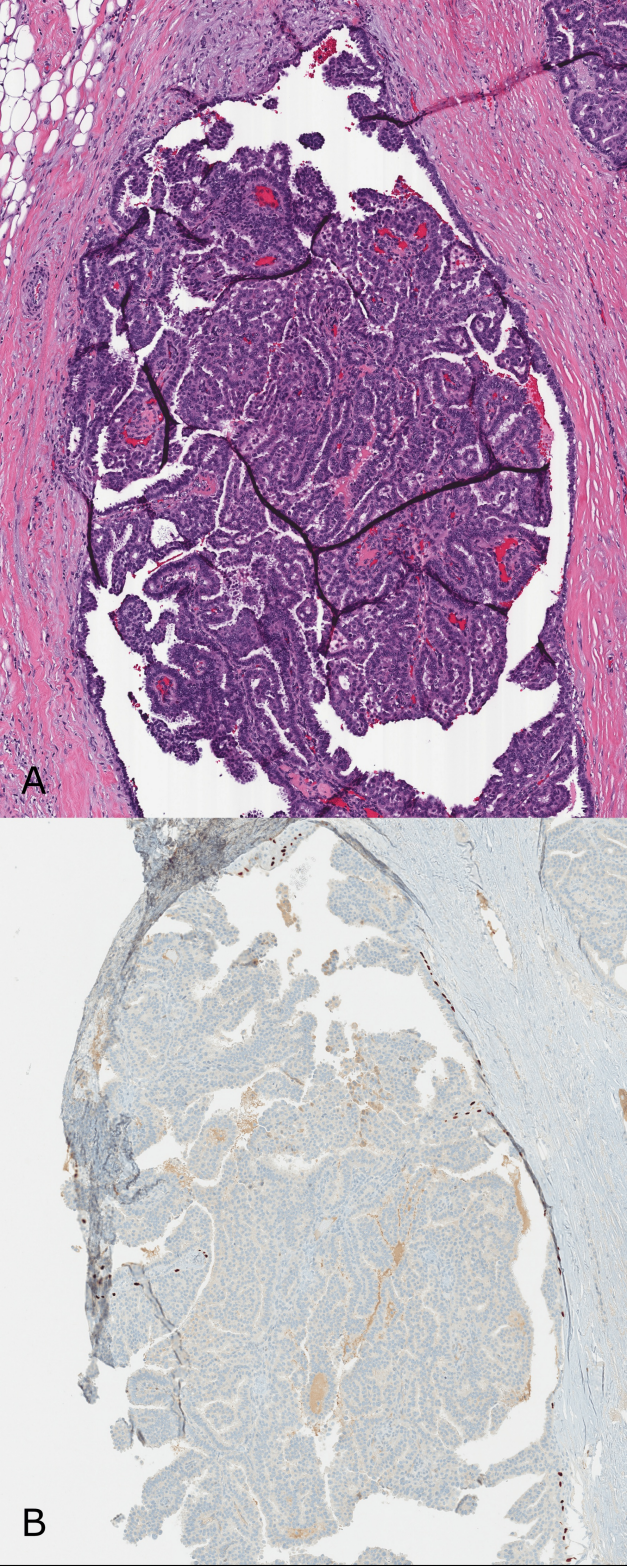
Area from the mastectomy specimen consistent with papillary DCIS. (A) H&E - High-power view of a papillary proliferation of cells with low to intermediate grade atypia. The fibrovascular cores are thin and delicate (40x). (B) p63 immunostain demonstrates nuclear staining in peripheral myoepithelial cells only, supporting papillary DCIS (40x).

**Figure 5:**
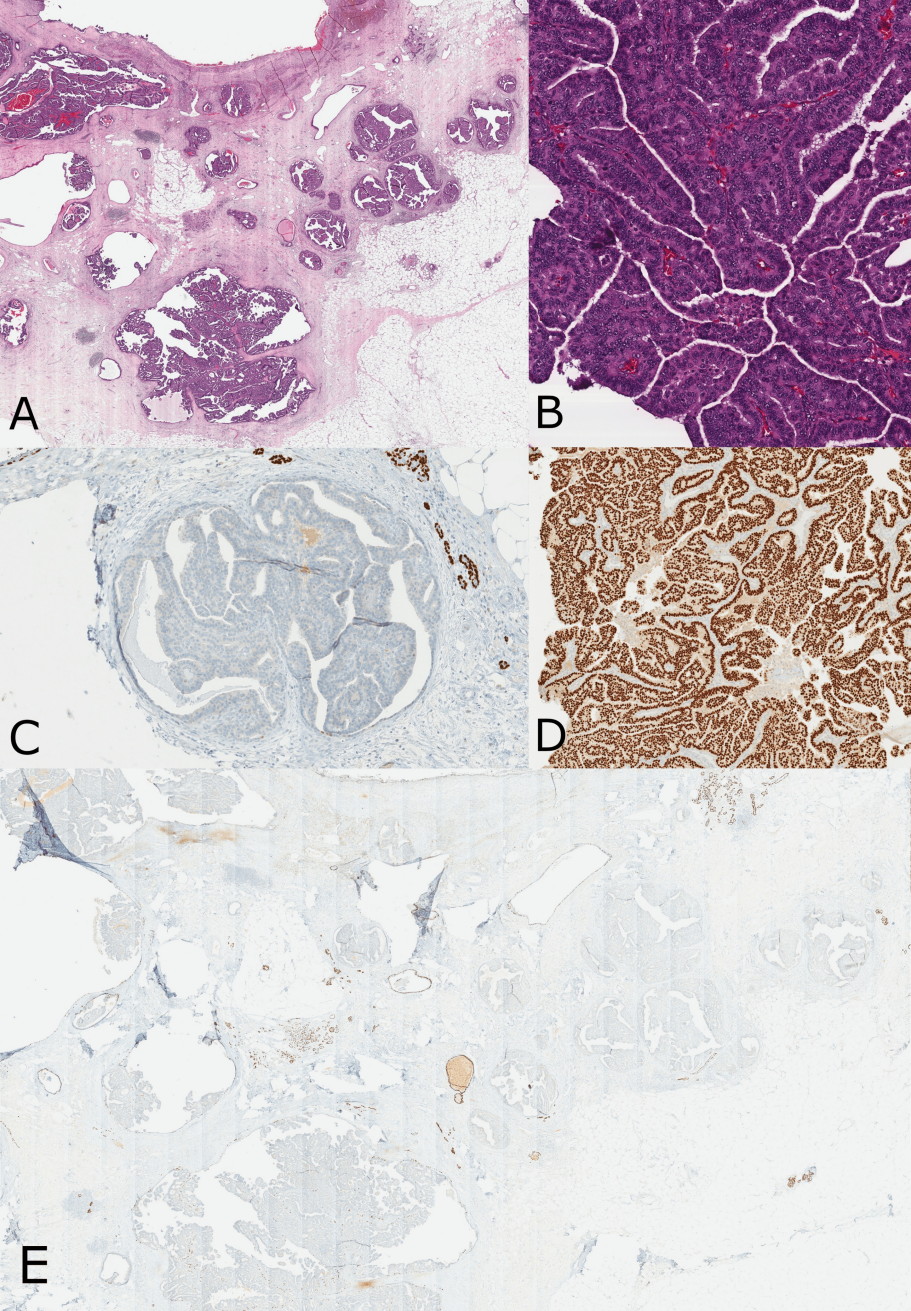
Area from the mastectomy specimen demonstrating an invasive carcinoma with multi-cystic papillary growth pattern. (A) H&E - The invasive component is composed of variably sized duct/cyst-like spaces with dense papillary architecture in the absence of a well-defined capsule. The invasive papillary structures infiltrate through and around normal breast elements (10x). (B) H&E - The lesion is composed of atypical epithelial cells with intermediate-grade nuclei lining papillary projections with well-defined fibrovascular cores (200x). (C) p63 immunostain is negative, demonstrating the absence of intralesional and peripheral myoepithelial cells and supporting the invasive nature (100x). (D) GATA3 immunostain strongly and diffusely stains the atypical nuclei, supporting primary breast pathology (100x). (E) At low power, p63 is negative within the invasive papillary cysts but highlights myoepithelial cells in normal breast glandular elements still present within the infiltrative lesion (10x).

**Figure 6:**
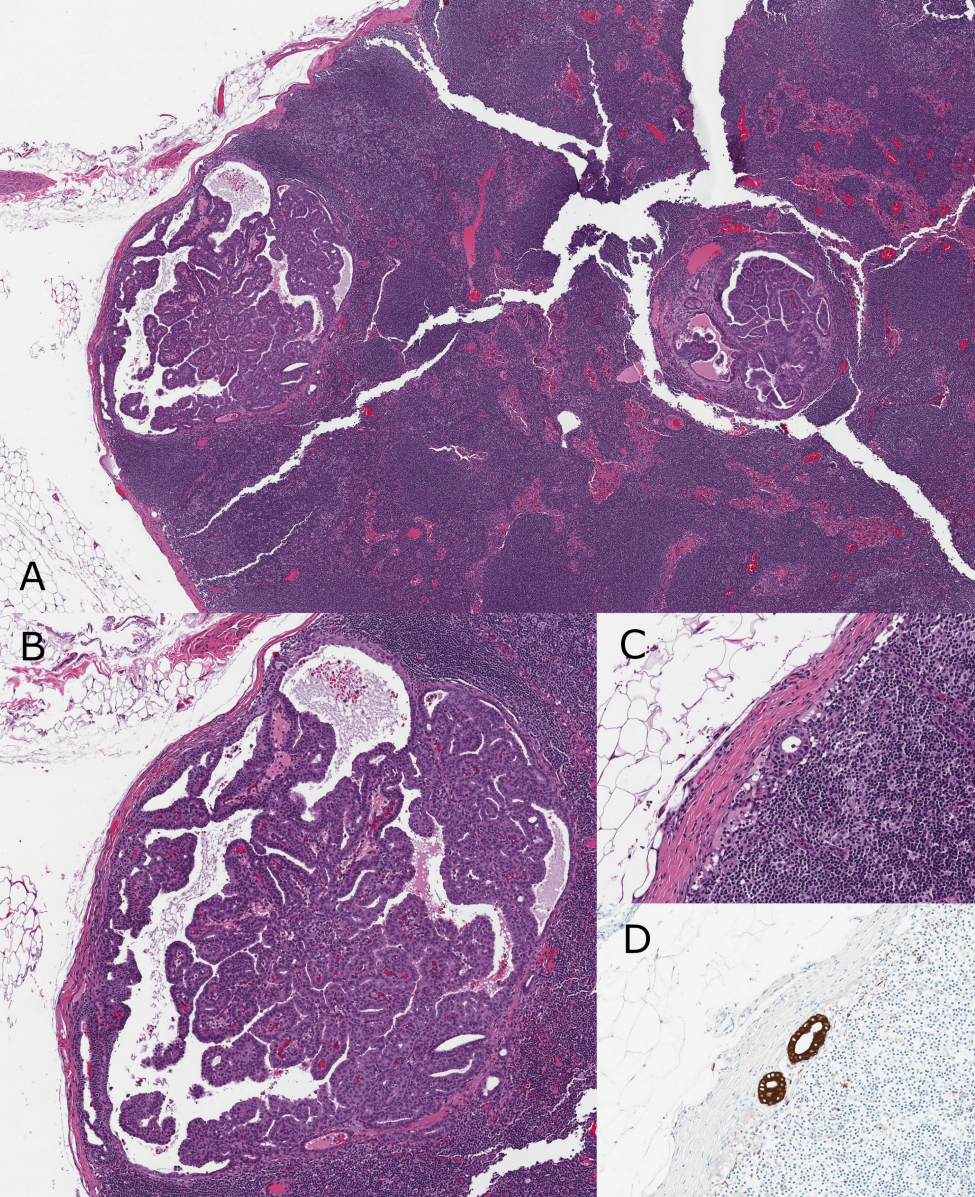
Lymph nodes demonstrating macrometastatic carcinoma and isolated tumor cells. (A) and (B) H&E - Macrometastatic (greater than 2 millimeters) carcinoma involving the sentinel lymph node. The carcinoma is morphologically equivalent to the breast primary carcinoma, demonstrating papillary architecture and low to intermediate-grade atypia (20x and 40x). (C) H&E and (D) Lu-5 immunostain of a non-sentinel lymph node demonstrating involvement by isolated tumor cells (fewer than 200 cells) (100x).

No neoadjuvant chemotherapy or radiation therapy was administered. Three months later, the patient underwent bilateral skin-sparing mastectomies with targeted right axillary sentinel node biopsy. Gross examination of the right mastectomy specimen revealed a firm, tan-brown mass with complex solid and cystic components measuring approximately 5.5 × 3.3 × 2.4 cm. Histologic examination of the mass demonstrated three distinct, concurrent papillary-type neoplastic processes. In the first process, atypical cells were present in areas with a papillary and cribriform architecture involving rounded ductal spaces with myoepithelial cells, as confirmed by p63 and calponin immunostaining, at the periphery and within fibrovascular cores, most consistent with atypia/DCIS involving a papilloma (Figure 3A,B). In the second process, atypia was also present in areas showing myoepithelial cells around the periphery but not within luminal fibrovascular cores, most consistent with papillary DCIS (Figure 4A,B). The third neoplastic process, the entity of interest for this case report, was an invasive carcinoma with a unique papillary growth pattern with intermediate-grade atypical cells forming duct/cyst-like spaces ranging in size from <1 mm up to 2.4 cm (Figure 5A,B). Myoepithelial markers (p63, calponin) were entirely negative (Figure 5C); there was no well-defined capsule, and the invasive component spanned a 5.5 cm area (Figure 5E). The lesion was given a Nottingham grade 2, with a score of 2 in each of the subcategories (glandular/tubular differentiation, nuclear pleomorphism, and mitotic rate). The patient’s targeted axillary biopsy yielded three total lymph nodes and was significant for a sentinel lymph node involved by radiologically occult macrometastatic disease with papillary architecture (Figure 6A,B) and one non-sentinel node involved by isolated tumor cells (Figure 6C, 6D). Both metastases were confirmed by cytokeratin (Lu-5) immunostain. The final pathologic stage was pT3pN1a. Additional synoptic data are compiled in Table 1. Hormonal biomarkers were repeated on the excision and were ER positive (91-100%) and PR positive (31-40%). HER2 testing by immunohistochemistry was negative (1+, which may be considered/reported “HER2 Low”), and the proliferation rate by Ki-67 immunostaining was 15-20% (luminal B). Oncotype diagnostics was performed, which resulted in a recurrence score of zero. Axillary dissection was deferred in lieu of adjuvant axillary radiation therapy. Upon completion of approximately one month of axillary radiation therapy, the patient began adjuvant endocrine (aromatase inhibitor) therapy.

**Table 1:** College of American Pathologists synoptic report DCIS: ductal carcinoma in situ, H&E: hematoxylin and eosin.

Case Summary: Invasive Carcinoma of the Breast	
Procedure	Total mastectomy
Specimen laterality	Right
Histologic type	Invasive carcinoma with papillary growth pattern
Glandular/tubular differentiation	Score 2
Nuclear pleomorphism	Score 2
Mitotic rate	Score 2
Overall grade	Grade 2
Tumor size	Greatest dimension of largest invasive focus: 55 mm
Tumor focality	Single focus of invasive carcinoma
Ductal carcinoma in situ (DCIS)	Present
Size (extent) of DCIS	At least 50 mm
Architectural pattern(s)	Cribriform, papillary, and micropapillary
Nuclear grade	Grade 2
Necrosis	Present, focal
Lobular carcinoma in situ	Not identified
Lymphatic and/or vascular invasion	Not identified
Dermal lymphatic and/or vascular invasion	Not identified
Microcalcifications	Present in DCIS and non-neoplastic tissue
Treatment effect in the breast	No known presurgical therapy
Margin status of invasive carcinoma	All margins negative for invasive carcinoma
Distance from invasive carcinoma to closest margin	Less than 1 mm
Closest margin to invasive carcinoma	Posterior
Margin status for DCIS	All margins negative for DCIS
Distance from DCIS to closest margin	Greater than 2 mm
Closest margin to DCIS	Posterior
Regional lymph node status	Tumor present in regional lymph nodes
Number of lymph nodes with macrometastases	1
Number of lymph nodes with micrometastases	0
Number of lymph nodes with isolated tumor cells	1
Size of largest nodal metastatic deposit	4 mm
Extranodal extension	Not identified
Total number of lymph nodes examined	3
Numer of sentinel nodes examined	3
pT Category	pT3
pN Category	pN1a
N Suffix	(sn)
Additional findings	Prior biopsy site changes, proliferative fibrocystic and fibroadenomatoid changes

## Discussion

We determined this case to be a primary breast cancer; the patient had no prior malignancy or radiographic evidence of any other primary malignancy. GATA3, an immunostain considered highly sensitive for carcinomas of breast origin, showed strong and diffuse nuclear staining [5].

This case exhibited a spectrum of papillary lesions. Some of the lesions fell within the well-described criteria for papillary DCIS with peripheral myoepithelial cells and papillary fronds lacking myoepithelial cells (Figure 4A, 4B). Other lesions fell within the criteria for atypia/DCIS within a papilloma, with myoepithelial cells both at the periphery and along their papillae (Figure 3A, 3B). Of interest, immunohistochemistry with p63 and calponin confirmed the complete absence of myoepithelial cells in a population of variably sized cystic, atypical papillary structures, which were dispersed in and around normal breast elements. Papillary neoplasms currently classified as completely devoid of myoepithelium include IPC, EPC, and SPC (which rarely show focal residual myoepithelial cells) [1]. However, these entities each have distinct histopathologic features that were not present in our case.

The WHO describes IPC as a single lesion with frankly infiltrating architecture, papillary morphology, and usually low to intermediate-grade nuclear atypia. Histologically, it is composed of mildly dilated ducts and microcysts containing papillary formations and completely lacks myoepithelium [4,6]. IPC is considered extremely rare and was often misdiagnosed (likely representing EPC, SPC, or a metastasis rather than a true IPC); consequently, there are limited data on it and few images to which we could compare our case [6]. Despite these limitations, we believe the WHO description of IPC does not accurately describe our case, which was composed of cysts up to 2.4 cm in size with papillary formations, without duct/tubule formation or microcysts. Our case also did not truly appear infiltrative, as required for a diagnosis of IPC, but exhibited rounded cysts scattered in and around normal breast elements, more like an intraductal pattern.

EPC is an uncommon (0.5-2.0%) subtype of breast cancer; EPC with invasion is even less common [7]. The WHO describes EPC as a papillary mass within a single cystic space or, less often, composed of an aggregate of close nodules with rounded borders, typically surrounded by a fibrous capsule of varying thickness. The papillary fronds are delicate and covered by low- to intermediate-grade nuclear epithelium. Micropapillary, solid, or cribriform architecture can sometimes be present. There are usually no myoepithelial cells present throughout the lesion [8]. When comparing these features to our case, it is notable that the papillary areas completely lacking myoepithelium in our case did not have a well-developed fibrous capsule. The borders of each cystic structure were rounded but blended inconspicuously with the adjacent stroma, without significant fibrosis (Figure 5A). Although the exact distance between cystic nodules to be considered “an aggregate of close nodules” is not defined by the WHO, we believe a reasonable assumption may be 1-2 mm, without or with minimal normal breast parenchyma in between them. Our case was composed of multiple cystic spaces, some greater than 2 mm apart, spanning an area of 5.5 cm, with a significant amount of normal breast parenchyma (composed of unremarkable lobules and stroma) within the lesional area (Figure 5A). These findings are not in keeping with the definition of EPC. EPC with frank invasion is another consideration; however, the frankly invasive component has never been described as having papillary morphology; rather, it is usually invasive ductal carcinoma of no special type [8]. Furthermore, the papillary carcinoma in this case was metastatic to lymph nodes, while only rare examples (0-3.7%) of pure EPC have metastases [9-11].

The WHO describes SPC as having a solid growth pattern with inconspicuous, delicate fibrovascular cores. The cells are monotonous, round to spindled, with low- to intermediate-grade nuclear atypia and eosinophilic granular cytoplasm, sometimes with mucin vacuoles. Myoepithelial cells are usually completely absent. It is considered in situ when composed of rounded nodules with an intraductal-type distribution pattern. It is considered invasive if composed of ragged-edged nodules creating a jigsaw pattern with desmoplastic stroma [12]. Though our papillary carcinoma had a similar distribution pattern to SPC in situ, it does not share many key architectural or cytologic features of SPC; it lacks a solid growth pattern, and the cells do not have eosinophilic granular cytoplasm.

## Conclusions

Per our review of the literature and the current WHO guidelines, we believe our case represents a distinct morphology not currently classified by the WHO. To our knowledge, this is the first reported invasive papillary-type breast carcinoma with these features, and we believe it to be either a distinct entity or a unique, unrecognized subtype of IPC or EPC. We speculate that the prognosis is likely comparable to that of invasive breast carcinoma of no special type, given the lymph node metastases at the time of presentation; however, further studies and case series are needed to determine the prognosis and management for this entity.
